# Proinflammatory role of monocytes in SARS-CoV-2 infection in chronic hemodialysis patients

**DOI:** 10.3389/fimmu.2023.1210961

**Published:** 2023-08-03

**Authors:** Viorica Bumbea, Luminita Ardelean, Luminita Radulescu, Luminita Damian, Horia Bumbea, Ion Dumitru, Claude Lambert, Ana-Maria Vladareanu

**Affiliations:** ^1^Department of Dialysis, Emergency Clinical Hospital Bucharest, Bucharest, Romania; ^2^Department of Nephrology, Emergency University Hospital Bucharest, Bucharest, Romania; ^3^Department Hematology, Emergency University Hospital Bucharest, Bucharest, Romania; ^4^University of Medicine and Pharmacy Carol Davila Bucharest, Bucharest, Romania; ^5^Department Immunology, Centre Hospitalier Universitaire (CHU) de Saint Etienne, Saint Etienne, France

**Keywords:** end-stage kidney disease (ESKD), chronic hemodialysis patients, HLA-DR, nonclassical monocytes, CD300E, intermediate monocytes

## Abstract

**Background:**

Fully mature monocytes that express CD14, but not CD16, undergo phagocytosis within tissues, whereas non-classical monocytes, CD14-low CD16+, represent <11% of peripheral monocytes and have primary pro-inflammatory functions. Inflammation plays a major role in Covid-19 disease and adds to the inflammation caused by chronic hemodialysis. The aim of our study was to monitor monocyte subsets in five patients with end-stage kidney disease (ESKD) over a 1-year period after a mild Covid-19 infection. Five ESKD patients with a mild Covid-19 infection were monitored using CD14, CD16, CD300e, HLA-DR, CD64, and CD45 panels using a BD FACS Canto flow cytometer.

**Results:**

CD14-low CD16+ was dramatically (p=0,001) decreased in patients during Covid-19 infection, as previously described for patients without chronic renal failure. In addition, CD14-low CD16+ monocytes remained decreased for 10 months after recovery from Covid. Intermediate monocytes increased during Covid-19 infection and decreased 10 months after infection but this subtype of monocytes retained their inflammatory activity with a significant increase in HLA-DR expression after recovery from Covid infection.

**Conclusion:**

Our study shows that ESKD patients had a pro-inflammatory profile induced by Covid 19, but this status was prolonged significantly over a 10-month period. Thus, advanced renal failure treated by hemodialysis did not dramatically change the inflammatory response against to SARS Covid 2. It seems that monocytes retain their inflammatory status for many months in ESKD patients after a Covid-19 infection.

## Introduction

Specific subsets of hematopoietic cells (described in the literature) have different functions in the immune response against various infections that involve an inflammatory response, and also have various consequences on other pathologies, such as cardiovascular diseases (1). The body’s defense against environmental factors is assured by physical barriers (skin, mucosa, etc.), by innate immunity (phagocytic cells like monocytes and macrophages, natural killer cells, complement) and adaptive immunity (B and T cells) (2). The effectiveness of the immunity is assured both by cells specialized in the defense of the body and soluble substances involved in inflammation (3). The role of the immune system is, on one hand, to detect and destroy germs, cancer cells, but also to repair tissues affected by injuries, infections, ischemia, toxins or autoimmunity (3).

Monocytes play a major role in acute (as observed in Covid-19) and chronic diseases (as observed in metabolic diseases associated with cardiovascular damage) (4, 5). Monocytes play a major role in acute (as observed in Covid-19) and chronic diseases (as observed in metabolic diseases associated with cardiovascular damage) (4, 5). In the case of a microbial stimul like viral, bacterial, protozoal or fungal pathogens the inflammatory monocytes react by secretion of cytokines and antimicrobial factors, migration to the site of microbial infection, expression of CCR2 chemokine receptor (6). It is how monocytes fight against Listeria monocytogenes, Mycobacterium tuberculosis, Toxoplasma gondii, Cryptococcus neoformans infection. Also inflammatory monocytes play a signifiant role in initiating and coordinating immune responses against viral infections such as influenza virus, respiratory syncytial virus or hantavirus infection (6–8)

During inflammation, monocytes can recognize and kill different pathogens. These antigen-presenting cells operate through HLA receptors and can also produce cytokines that amplify the immune response. This forms one path within the development of systemic inflammatory response syndrome (SIRS), a proinflammatory syndrome that operates to destroy pathogens. However, compensatory anti-inflammatory response syndrome (CARS) is a protective system that restores the organism’s homeostasis. It can exist separately from SIRS and can reverse inflammation. CARS induces a decrease in cytokine production and a decrease in HLA-DR receptors on monocytes (9).HLA-DR is part of the major histocompatibility complex (MHC) class II. This marker is common in the membrane of antigen-presenting cells, dendritic cells, macrophages, B lymphocytes, and activated T lymphocytes (10).

A decrease in the expression of the antigen-presenting molecules (human leukocyte antigen- HLA-DR) from monocyte membranes creates a protective response against inflammation. This decrease occurs immediately after strong activation of the immune system. If this decrease is severe, the response leads to immunodepression but also, secondarily, to elevated morbidity and mortality. A decrease in HLA-DR is an indicator for the presence of CARS. If the decrease persists below 60%, the immune response is termed immunodepression; if the decrease is below 30%, it is termed immuno-paralysis. If HLA-DR rapidly returns to >75% during injury, this is a marker for a good recovery (11).

Monocytes have typical immuno-phenotypic characteristics. These surface markers have been classified in the CD nomenclature system (approximately 250 different protein structures). A CD structure can act as a receptor or as a receptor activation site, their activation launching a waterfall of reactions belonging to the immune system. Some CD structures have a role in the cellular adhesion. There are surface CD markers specific to a certain cell, or that appear during a certain phase of its development, so that cells can be differentiated both as type and activity level or evolutive stage (12)

Various surface markers have been identified by flow cytometry: CD13, CD33, CD11b-CD18, CD4, CD64, and HLA-DR (13). Monocytes can express at any stage of maturation of CD4, CD33, and CD64. Immature monocytes express HLA-DR surface markers, but CD13 and CD14 are not expressed until the monocyte matures. In the activated mature monocyte, an increase of HLA-DR expression is also found (13). Antigen HLA-DR surface expression of monocytes reflects the activation state of these cells. If HLA-DR decreases during chronic stimulation, this can be an indicator of immunosuppression (14).

Monocytes can have pro- or anti-inflammatory activity, and this activity is reflected through the expression of surface HLA-DR. A decrease in HLA-DR is an immunosuppression marker and can be induced by sepsis. Conversely, a strong stimulation of monocytes and an increase in HLA-DR expression can occur in cytokine-release syndrome (15). A decrease in HLA-DR is commonly associated with a decrease in the number of non-classical monocytes (proinflammatory monocytes) (15).

The monocyte CD14+, associated with a decrease in the expression of human leukocyte antigen class II (HLA-DR), is a marker for immunodeficiency in some conditions, such as: trauma, major surgery, burns, sepsis, pancreatitis. This decrease is correlated with a poor outcome and/or mortality (16). However, if there is a decrease in the CD16+ monocyte HLA-DR during sepsis, this decrease seems to be transitory and less severe (16).

CD300 surface receptors modulate the immune-activation pathways involved in viral infections and sepsis, and release proinflammatory cytokines. The CD300 receptor family includes both activating and inhibitory receptors that develop the immune response. The receptors CD300: i.e., a, b, c, d, e, f, h, are type I transmembrane proteins found in lymphoid and myeloid cells. CD300a and CD300f isoforms are inhibitory receptors, whereas CD300b, CD300c, CD300d, CD300e, and CD300h are activating receptors (17).

CD300e appears on monocyte membranes, especially on CD14+ cells and on circulating myeloid dendritic cells (18). Activation of CD300e induces Ca^2+^ mobilization, a release of reactive oxygen species, and a release of cytokines. It plays a role in monocyte survival and also contributes to the activation of T cells (15, 19). CD300e, considered a receptor responsible for activating immunity, can be used to evaluate the number of activated monocytes (19–21).

Three subtypes of monocytes that use expression levels of CD14 and CD16 on the surface membrane have been described (22):

1. Classical monocytes, CD14+ and CD16-, represent approximately 80–85% of total monocytes. They are involved in migration to the site of inflammation, transformation into macrophages, and phagocytosis (23). They play an essential role in immune mechanisms for defense against microbial pathogens (4). This subset also has a pro-inflammatory role in chronic diseases like atherosclerosis, cancer, and rheumatoid arthritis (24–27).

2. A subset of “non-classical” monocytes express low levels of CD14 and strong levels of CD16. They represent 2-11% of peripheral monocytes (23, 28).

CD14-low CD16+ monocytes play a role in inflammation and antigen presentation (23). They are also involved in the formation of granulation tissue and in the detection of virally infected cells. This subset is also involved in the removal of dying cells, and of viruses and tumor cells from the circulation (4, 24, 29–31)). The number of non-classical monocytes play a role in metabolic syndrome and are positively correlated with total cholesterol, LDL cholesterol and triglycerides, and negatively with HDL cholesterol (4).

3. Intermediate monocytes CD14+ and CD16+, which represent 2-8% of monocytes, have a dual role in inflammation and phagocytosis (23, 28). This subtype of monocyte occurs in large numbers in inflammatory diseases, reaching up to 50% in cases of sepsis in some studies (4, 32, 33). A high number of intermediate monocytes occur in the presence of inflammation and cytokines (34–36) and can be considered an independent predictive marker for a cardiovascular event (4).

In some reports, both intermediate and non-classical (CD16+) monocytes can play a significant role in atherosclerosis processes and have been correlated with atherosclerotic plaque in patients with angina pectoris (especially patients with ESKD) (4, 37). The importance of monocyte subtypes in septic and inflammatory diseases has been demonstrated in patients with systemic lupus erythematous and in sepsis: this helps confirm the hypothesis that CD16+ subtypes of monocytes are involved mainly in inflammation, as based on the high percentage found in inflammatory diseases (38).

Inflammation is an important risk factor for cardiovascular disease which is the most important cause of mortality in dialyzed patients.

The number of monocytes has been correlated with endothelial damage (24). CD16+ monocytes are elevated in chronic hemodialyzed patients and intermediate monocytes may be a predictor of their cardiovascular morbidity (4).

ESKD, together with cardiovascular disease and diabetes, have been considered as comorbidities for severe Covid-19 disease (39)

Monocytes are considered to play an important role in immunopatology and disease severity in Covid-19. Patients with medium forms of Covid 19 infection presents an increase of activated intermediate monocytes following an early anitiviral response in the nosopharynx (8). In the case of viral infection, like Covid 19 infection, monocytes migrate in the affected tissue (in our case respiratory tract) to defend against pathogen. In respiratory tract monocytes transform themselves in inflammatory macrophages and gain effector functions of pro- and anti-inflammatory activities, antigen-presentation and tissue remodeling (40). In the lung, macrophages (both interstitial and alveolar) are the most abundant immune cells. The activation of alveolar monocytes leads to a high phagocytic capacity, higher oxidative burst and increased release of pro-inflammatory cytokines and chemokines. It result inflammation and migration of other inflammatory cells in lung. If the inflammation is prolonged and disregulated, tissue damage can occur. To prevent persistent inflammation, alveolar monocytes act through phagocytosis of dying cells and release of TGFβ, IL-10, prostaglandin E2 and platelet-activating factor. Chronic conditions like asthma or chronic obstructive pulmonary disease present a lack in phagocytic activity leading to persistent inflammation (40).In this study, we assessed the variable expression of different markers on the monocyte membranes of dialyzed patients infected by SARS-CoV-2 and assessed if Covid-19 influenced the distribution of subtypes of monocytes. The aim of this study was to monitor the immune phenotype of ESKD patients with mild Covid-19.

## Materials and methods

This prospective observational study was started in 2020. It included patients admitted into the Hemodialysis Department of the Emergency Hospital in Bucharest. Changes in immunity in uremic patients treated with dialysis procedures were assessed. We enrolled 15 stable chronic dialysis patients with no serious comorbidities. No patient had diabetes, nor a major cardiovascular event (such as a history of acute myocardial infarction or stroke), and no recent history of an infection. In the event hypertension condition was present it had to be well controlled with medication and hemodialysis. Drugs used for hypertension was beta blockers, converting enzyme inhibitor and calcium blocker. No additional antihypertensive medication was necessary during the study.These patients were compared with a control group of healthy subjects with no renal failure (with normal GFR). There were 12 healthy subjects and 15 hemodialysed patients, with a mean age of 62,3 for the healthy ones and 67,8 for the hemodialysed ones. 7 subjects from the healthy group and 10 from the hemodialysed group were women (**Table 1**). We performed analysis by flow-cytometry on T-cells, B-cells, and monocyte subsets taken from peripheral blood. This study started just before the onset of the Covid-19 pandemic in our country.

**Table 1:** Age and sex ratio distribution of patients enrolled in the initial study.

	Healthy subjects	Hemodialyzed patients
*number*	12	15
*women*	7 (58%)	10 (67%)
*men*	5 (42%)	5 (33%)
*Mean of age*	62,3	67,8

Seven of the 15 ESKD patients studied caught Covid-19 infection during the follow-up. Before vaccination was available, five (four females, one male), mean age 62 years (range: 36–82 years), of our ESKD patients had symptomatic Covid-19. Patients had been under chronic hemodialysis for 1-6 years consecutive to interstitial nephropathy (*n*=3), glomerulonephritis (*n*=1), or glomerulosclerosis (*n*=1*)*. Covid 19 was always mild or moderate, according to the guidelines, with fever, cough, malaise, headache, loss of taste and smell, nausea with respiratory symptoms, but with saturation of oxygen ≥94% in room air (41). They all recovered within a few days under symptomatic treatment, patients received acetaminophen, ibuprofen, ambazone (oral antiseptic) acetylcysteine in the case of productive cough and herbal cough suppressants like Calmotusin or Antitusin (they are considered food supplement). Monitoring was performed at the time and at a mean of 10 months after the Covid infection: i.e., one patient after 3 months, two patients after 11 months, and 2 patients after 13 months. Hemodialysed patients were tested every 2 weeks during pandemia or if they presented simptoms: fever, cough, rhinorhea. The sample was harvested immediately after the positive result was observed, at the next dialysis session, after 2 days (**Table 2**).

**Table 2:** Description of timelines of samples collection for study patients.

	First sample	Secondary sample-Covid infection	Secondary sample-Covid infection	Secondary sample-Covid infection	Secondary sample-Covid infection	Secondary sample-Covid infection	Last semple
**1**	7/28/2020	11/18/2020					12/08/21
**2**	7/30/2020		11/23/2020				12/08/21
**3**	7/30/2020			1/6/2021			12/08/21
**4**	7/30/2020				1/20/2021		12/08/21
**5**	7/30/2020					9/7/2021	12/08/21

After obtaining informed consent from each subject, a blood sample (~2 mL) was taken on EDTA and subsequently processed using the simple Lyse-Wash protocol: CD14, CD16, CD300e, HLA-DR, CD64, and CD45 (complete with fluorochromes and providers), and was analyzed with Gallios software (Navios™) (Beckman-Coulter; Fullerton, CA).

Collection of peripheral blood samples was performed by intravenous puncture and then placed in tubes with EDTA. Samples were kept at room temperature until transported to the laboratory. Approximately 100 μL of blood was taken from each sample and placed in a Falcon tube together with 5–20 μL of labelled monoclonal antibodies. The samples were vortexed, incubated for 15 min in the dark at room temperature, the red blood cells were lysed, and then the tubes were inserted into the cytometer for data acquisition. We used the Gallios flow-cytometer, produced by Becton Dickinson, which was equipped with three lasers and 10 colors. For the study of monocytes, antibodies were used to identify the markers: CD14, CD16, CD300e, HLA-DR, CD64, and CD45. Monoclonal antibodies from the following manufacturers were used: Beckman-Coulter, Immunotech (BD Biosciences^®^ San Jose, CA), BD Pharmingen, Dako, Cytognos, CD14, APCH7, MΦP9, BD; CD16, FITC, CLB-FcGran1, BC; CD300e, APC, IREM-2, Immunotech; HLA-DR, V450, L243, BD; CD64, PE, 10,1, BD; CD45, OC515, HI30, Immunostep.

The Shapiro–Wilk test was used to determine if the variables had a normal distribution. If there was no exception, a paired t-test was used. If the data deviated from normality, then the Wilcoxon test was applied using Jasp and NCSS software. Statistically significant differences were considered when *p*<0.05. Our cohort was small, but the results were obtained using paired analysis, thus with greater statistical power to assess the interval between the first and second blood collections (on average a 7-month interval), and a third collection at month 10. No new events occurred during that time that added confounding factors.

## Results

The five patients with moderate Covid infection included in the study were four females and one male with a mean age of 62 years. They all had a moderate form of Covid-19 (including fever, cough, malaise, headache, loss of taste and smell, nausea with respiratory symptoms, but with saturation of oxygen ≥94% in room air). The mean time on dialysis was 3 years at commencement of the study. The mean time between the first and the second sample was 6.4 months; the third test was conducted at 10 months after Covid-19 infection.

The patients did not receive any treatment for any infection other than Covid-19. All our patients received symptomatic treatment with antipyretic and antiemetic drugs, and vitamins during the Covid infection.

**Table 3** show the descriptive statistics for white blood cells, monocytes and lymphocyte absolute counts, during, and after Covid infection.

**Table 3:** Descriptive statistics and paired t test of WBC, lymphocytes and monocytes values before, during and after Covid infection.

Samples to compare	Mean	SD	Samples to compare	Mean	SD	p
Leukocytes before Covid infection	9120	2385,7	Leukocytes during Covid infection	5426	2264	0.023
Leukocytes during Covid infection	5426	2264	Leukocytes after Covid infection	7290	1432	0.206
Leukocytes before Covid infection	9120	2385,7	Leukocytes after Covid infection	7290	1432	0.109
Lymphocytes before Covid infection	1616	948,8	Lymphocytes during Covid infection	1042	723,1	0.029
Lymphocytes during Covid infection	1042	723,1	Lymphocytes after Covid infection	1358	416,9	0.311
Lymphocytes before Covid infection	1616	948,8	Lymphocytes after Covid infection	1358	416,9	0.504
Monocytes before Covid infection	646	365,2	Monocytes during Covid infection	368	194,2	0.167
Monocytes during Covid infection	368	194,2	Monocytes after Covid infection	766	477,9	0.212
Monocytes before Covid infection	646	365,2	Monocytes after Covid infection	766	477,9	0.504

In order to see the differences between the three subtypes of monocytes we analyzed the percentage of classical monocytes with the expression of CD300e and HLA-DR (**Table 4**), non-classical monocytes with the expression of CD300e and HLA-DR (**Table 5**), and intermediate monocytes with the expression of CD300e and HLA-DR (**Table 6**) at before, during and after Covid infection

**Table 4:** Descriptive statistics and paired t test of classical monocyte percentages with expression for CD300e and expression of HLA-DR before, during and after Covid infection.

Samples to compare	Mean	SD	Samples to compare	Mean	SD	p
Classical monocytes before Covid infection	73,4	9,6	Classical monocytes during Covid infection	59	28,9	0.295
Classical monocytes during Covid infection	59	28,9	Classical monocytes after Covid infection	86,6	2,1	0.113
Classical monocytes before Covid infection	73,4	9,6	Classical monocytes after Covid infection	86,6	2,1	0.057
Classical monocytes CD300e before Covid infection	94,6	1	Classical monocytes CD300e during Covid infection	89,4	8,5	0.240
Classical monocytes CD300e during Covid infection	89,4	8,5	Classical monocytes CD300e after Covid infection	91,8	7,7	0.151
Classical monocytesCD300e before Covid infection	94,6	1	Classical monocytes CD300e after Covid infection	91,8	7,7	0.454
Classical monocytes HLADR before Covid infection	71,6	9	Classical monocytes HLADR during Covid infection	75,2	21,9	0.745
Classical monocytes HLADR during Covid infection	75,2	21,9	Classical monocytes HLADR after Covid infection	81,1	7,7	0.650
Classical monocytes HLADR before Covid infection	71,6	9	Classical monocytes HLADR after Covid infection	81,1	7,7	0.201

**Table 5:** Descriptive statistics and paired t test of non-classical monocyte percentages with expression of CD300e and expression of HLA-DR before, during, and after Covid infection.

Samples to compare	Mean	SD	Samples to compare	Mean	SD	p
Nonclassical monocytes before Covid infection	8,2	3,2	Nonclassical monocytes during Covid infection	1,8	1,7	0.001
Nonclassical monocytes during Covid infection	1,8	1,7	Nonclassical monocytes after Covid infection	0,8	0,6	0.361
Nonclassical monocytes before Covid infection	8,2	3,2	Nonclassical monocytes after Covid infection	0,8	0,6	0.010
Nonclassical monocyteCD300e before Covid infection	58,4	26,2	Nonclassical monocyteCD300e during Covid infection	61,6	22,4	0.654
Nonclassical monocyteCD300e durind Covid infection	61,6	22,4	Nonclassical monocyteCD300e after Covid infection	36,2	15,3	0.077
Nonclassical monocyteCD300e before Covid infection	58,4	26,2	Nonclassical monocyteCD300e after Covid infection	36,2	15,3	0.082
Nonclassical monocytes HLADR before Covid infection	20,9	11,4	Nonclassical monocytes HLADR during Covid infection	64	33,7	0.033
Nonclassical monocytes HLADR during Covid infection	64	33,7	Nonclassical monocytes HLADR after Covid infection	9,6	8,2	0.023
Nonclassical monocytes HLADR before Covid infection	20,9	11,4	Nonclassical monocytes HLADR after Covid infection	9,6	8,2	0.087

**Table 6:** Descriptive statistics and paired t test of intermediate monocyte absolute percentages with expression of CD300e and expression of HLA-DR before, during, and after Covid infection. .

Samples to compare	Mean	SD	Samples to compare	Mean	SD	p
Intermediate monocytes before Covid infection	11,2	4,8	Intermediate monocytes during Covid infection	37	28,8	0.115
Intermediate monocytes during Covid infection	37	28,8	Intermediate monocytes after Covid infection	6,3	1	0.077
Intermediate monocytes before Covid infection	11,2	4,8	Intermediate monocytes after Covid infection	6,3	1	0.049
Intermediate monocytesCD300e before Covid infection	96,2	3	Intermediate monocytesCD300e during Covid infection	95,2	3,8	0.690
Intermediate monocytesCD300e during Covid infection	95,2	3,8	Intermediate monocytesCD300e after Covid infection	97	1,5	0.244
Intermediate monocytesCD300e before Covid infection	96,2	3	Intermediate monocytesCD300e after Covid infection	97	1,5	0.517
Intermediate monocytes HLADR before Covid infection	68,7	7,9	Intermediate monocytes HLADR during Covid infection	80,2	18	0.332
Intermediate monocytes HLADR during Covid infection	80,2	18	Intermediate monocytes HLADR after Covid infection	87,8	4,7	0.416
Intermediate monocytes HLADR before Covid infection	68,7	7,9	Intermediate monocytes HLADR after Covid infection	87,8	4,7	0.012

**Figure 1:**
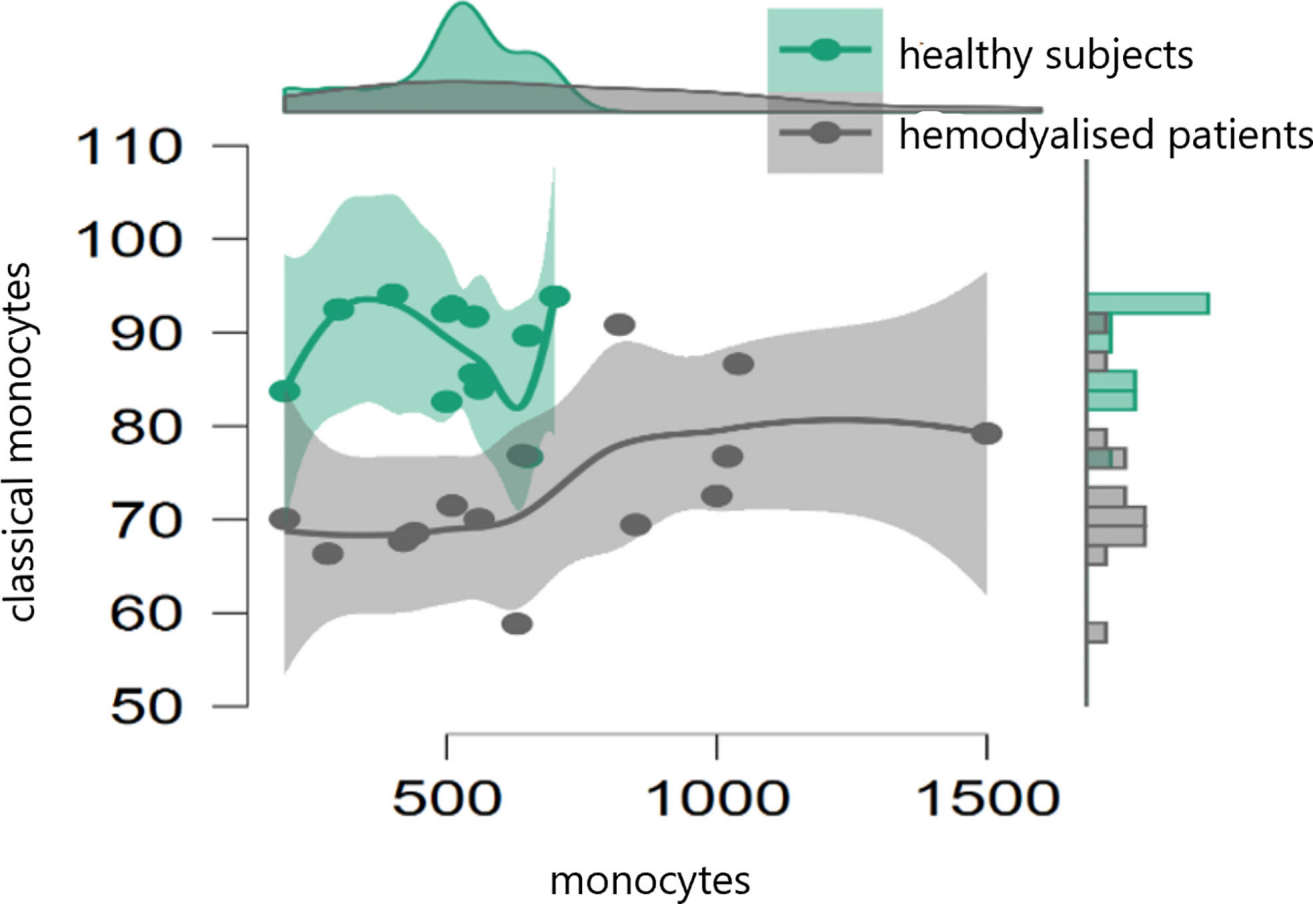
Dot plot histogram representing the percentage of classical monocytes lower in dialyzed patients compared to the control group (p:0,001).

**Figure 2:**
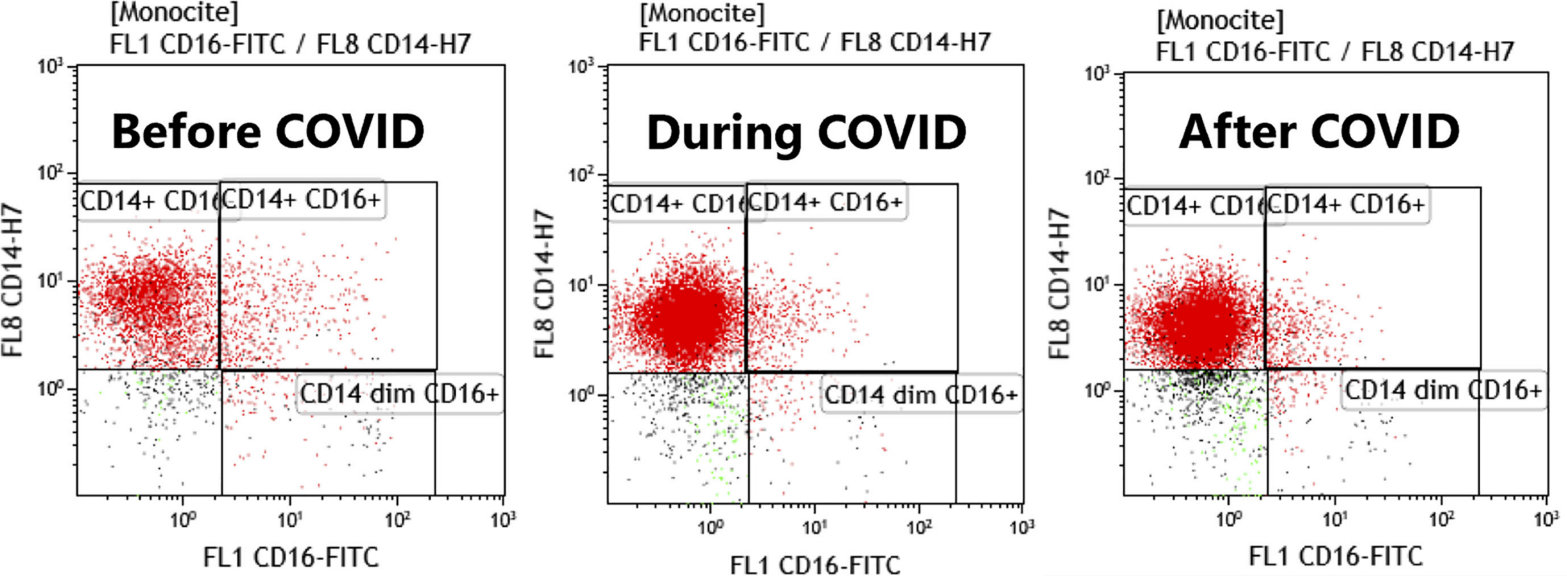
Dot plot histogram representing the percentage of non-classical monocytes elevated in dialyzed patients compared to the control group (p:0,001).

**Figure 3:**
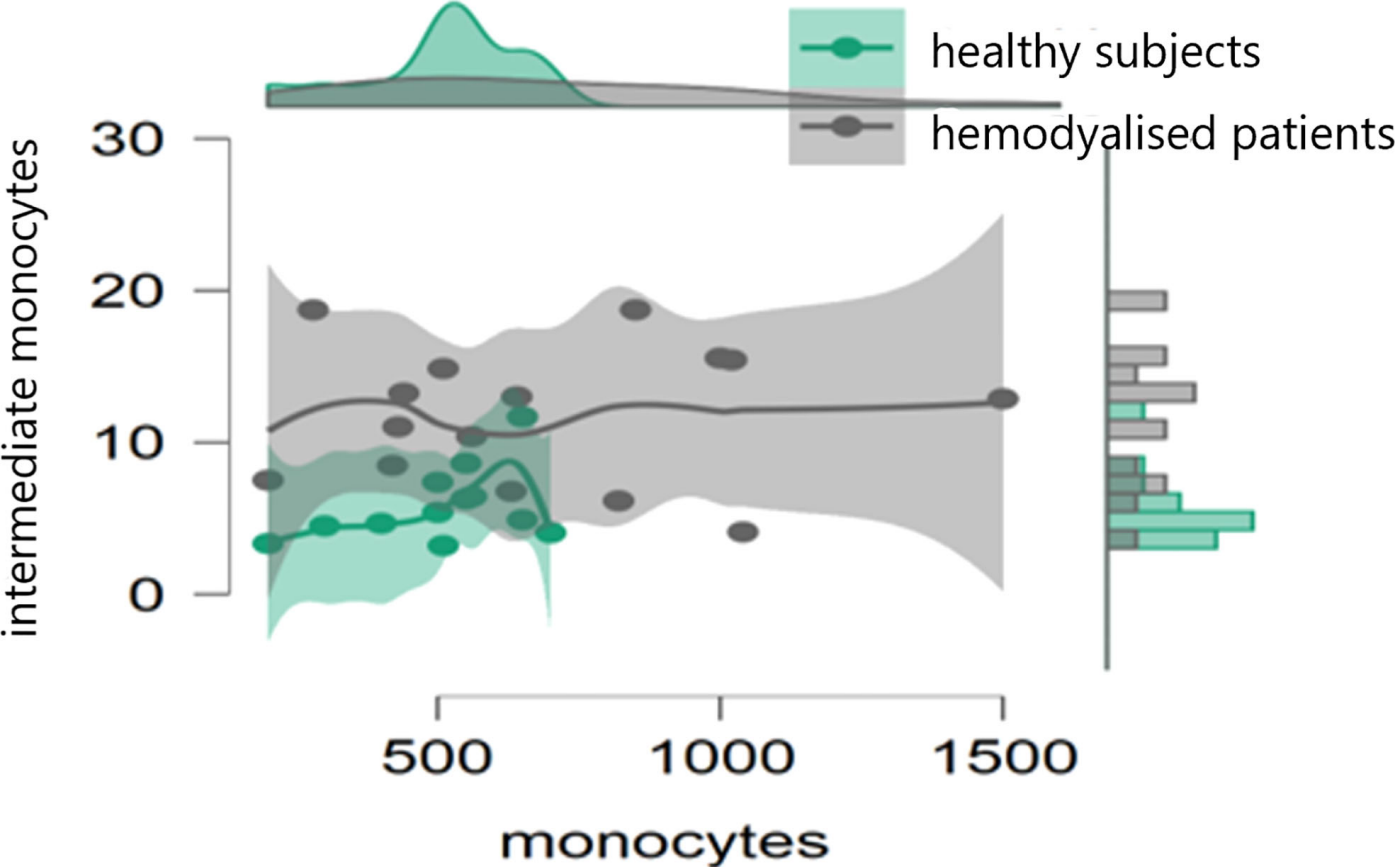
Dot plot histogram representing the percentage of intermediate monocytes elevated in dialyzed patients compared to the control group (p:0,001).

In the initial study (on 15 hemodialyzed patients with no Covid infection versus control) monocytes were obtained with the following results: no differences were found between the number of monocytes and leucocytes in hemodialyzed patients versus the healthy controls. The percentage of classical monocytes was lower in dialyzed patients compared to the control group (p:0,001, **Figure 1**) Both non-classical (p:0,001, **Figure 2**) and intermediate monocytes (p:0,001, **Figure 3**) were significantly elevated in dialyzed patients compared to the control group, and there was increased expression of HLA-DR and CD300e on CD16+ monocyte in ESKD patients (**Table 7**).

**Table 7:** Descriptive statistics and paired t test of non-classical monocyte percentages with expression of CD300e and expression of HLA-DR between healthy subjects and patients.

Samples to compare	Mean	SD	Samples to compare	Mean	SD	p
Monocytes healthy subjects	505,8	145,6	Monocytes hemodialyzed patients	689,3	347,7	0,1
HLADR monocytes healthy subjects	75,8	15,5	HLADR monocytes hemodialyzed patients	69,9	15,6	0,336
CD300e monocytes healthy subjects	85,2	9,4	CD 300e monocytes hemodialyzed patients	88,4	5,4	0,5
Classical monocytes healthy subjects	88,2	5,5	Classical monocytes hemodialyzed subjects	72,9	8,1	0,001
Classical HLADR monocytes healthy subjects	77,2	13,3	Classical HLADR monocytes hemodialyzed subjects	78,3	12,3	0,9
Classical CD300e monocytes healthy subjects	87,7	10,6	Classical CD300e monocytes hemodialyzed subjects	93,2	4,5	0,13
Nonclassical monocytes healthy subjects	1,06	1,2	Nonclassical monocytes hemodialyzed subjects	4,7	3,1	0,001
Nonclassical HLADR monocytes healthy subjects	43	20,6	Nonclassical HLADR monocytes hemodialyzed subjects	38,6	36,1	0,5 (0,001 for absolute values)
Nonclassical CD300e monocytes healthy subjects	57,5	61,5	Nonclassical CD300e monocytes hemodialyzed subjects	63,2	22,4	0,4
Intermediate monocytes healthy subjects	5,8	2,4	Intermediate monocytes hemodialyzed subjects	11,7	4,5	0,001
Intermediate HLADR monocytes healthy subjects	89,2	6,5	Intermediate HLADR monocytes hemodialyzed subjects	82,3	12,2	0,09 (0,01 for absolute value)
Intermediate CD300e monocytes healthy subjects	96,2	2,7	Intermediate CD300e monocytes hemodialyzed subjects	97,3	2,3	0,2 (0,001 for absolute value)

**Figure 4:**
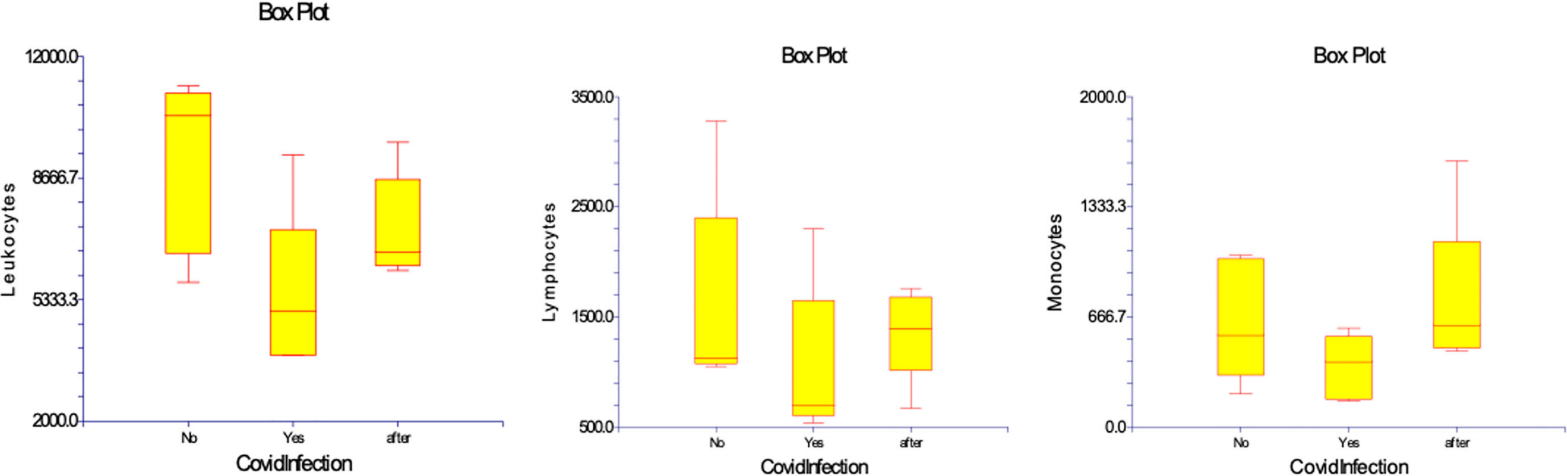
Numbers of WBC, lymphocytes, and monocytes in patients before, during and after Covid-19 infection. Results are presented in box plots indicating the median as the horizontal line, 25^th^-75^th^ percentiles as the group distributions represented as boxes, and 2.5--97.5% cumulative frequencies as shown as whiskers. Outliners [identified by the 1.5 x inter-quartile range (IQR) criterion] are plotted as empty squares. * represents p<0.05 and ** represents p<0,01.

In the study regarding the five patients with Covid infection the results did not differ in terms of hematocrit and hemoglobinemia during the evolution. Leucocytosis significantly declined from 9,120 (5,800 – 11,200) cell/µL before Covid-19 infection in acute Covid-19 to 5,426 (3,800 – 9,300) cell/µL (*p*= 0,023). This was mainly related to lymphopenia 1,042 (540 – 2,300) cell/µL, as compared to values before Covid at 1,616 (1,050 – 3,280) cell/µL (*p*=0,029), whereas monocytes were not significantly changed 368 (160 – 600 cell/µL) as compared to 646 (200 – 1,040) cell/µL (*p*= 0,167). At 10 months after Covid-19 recovery, lymphocyte counts were still low at 1,358 (670– 1,760) cell/µL, whereas monocyte numbers were within same range. (**Table 3**) (**Figure 4**)

**Figure 5:**
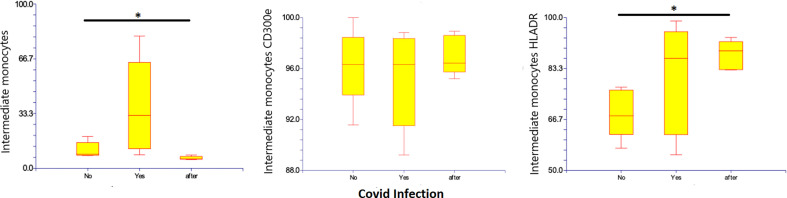
Non-classical CD14-low CD16+ subsets of monocytes, expression of percentage and expression of CD 300e and HLA-DR on non-classical CD14-low CD16+ subsets of monocytes before, during, and after COVID-19 infection. Results are presented in box plots indicating the median as the horizontal line, 25^th^-75^th^ percentiles as the group distributions as boxes, and 2.5-97.5% cumulative frequencies as whiskers. Outliners [identified by the 1.5 x inter-quartile range (IQR) criterion] are plotted as empty squares. * represents p<0.05 and ** represents p<0,01.

CD14-low CD16+ monocytes were significantly reduced in patients with Covid-19, representing 1.8% (0.5 – 4.9%) of monocytes (*p*=0.010), as compared to those with non-infected ESKD: 8.27% (6.2 – 13.9%) of monocytes. Their numbers remained low at 10 months after recovery: 0.85% (0.29 – 1.9% of monocytes). Pro-inflammatory marker HLA-DR was more expressed on CD14-low CD16+ monocytes in Covid-19 at 64.06% (8.92 – 91.84% of non-classical monocytes) as compared to non-infected ESKD patients at 20.97% (6.37-36.17%), whereas expression of CD300e was not significantly changed: 61.6% (26.1 – 88.3% of non-classical monocytes) compared to 58.4% (18.3 – 85.5% of non-classical monocytes before Covid infection). (**Table 5**) (**Figure 5**)

**Figure 6:**
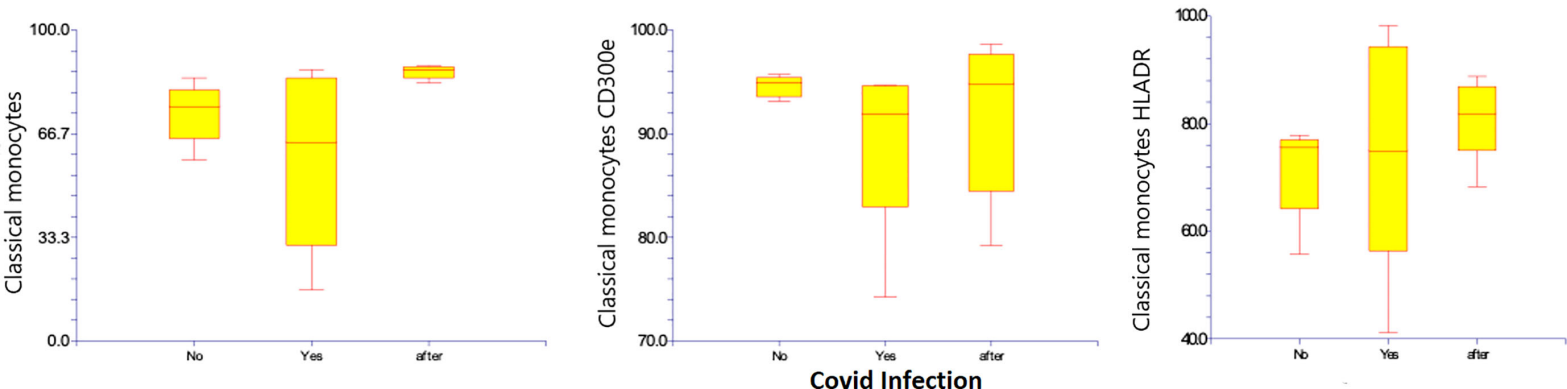
Classical CD14+ CD16- subsets of monocytes, and percentages and expression of CD 300e and HLA-DR on classical CD14+ CD16- subsets of monocytes before, during, and after Covid-19 infection. Results are presented in box plots indicating the median as the horizontal line, 25^th^-75^th^ percentiles as the group distributions are shown as boxes, and 2.5-97.5% cumulative frequencies are shown as whiskers. Outliners [identified by the 1.5 x inter-quartile range (IQR) criterion] are plotted as empty squares. * represents p<0.05 and ** represents p<0,01.

Regarding classical monocytes, neither HLA-DR 75.3% (41.2 – 98.2%) compared to 71.6% (55.7 – 77.8%), nor CD300e at 89.4% (74.3 – 94.7%) compared to 94.6% (93.1 – 95.7%) was significantly changed regarding classical monocytes. (**Table 4**) (**Figure 6**)

**Figure 7:**
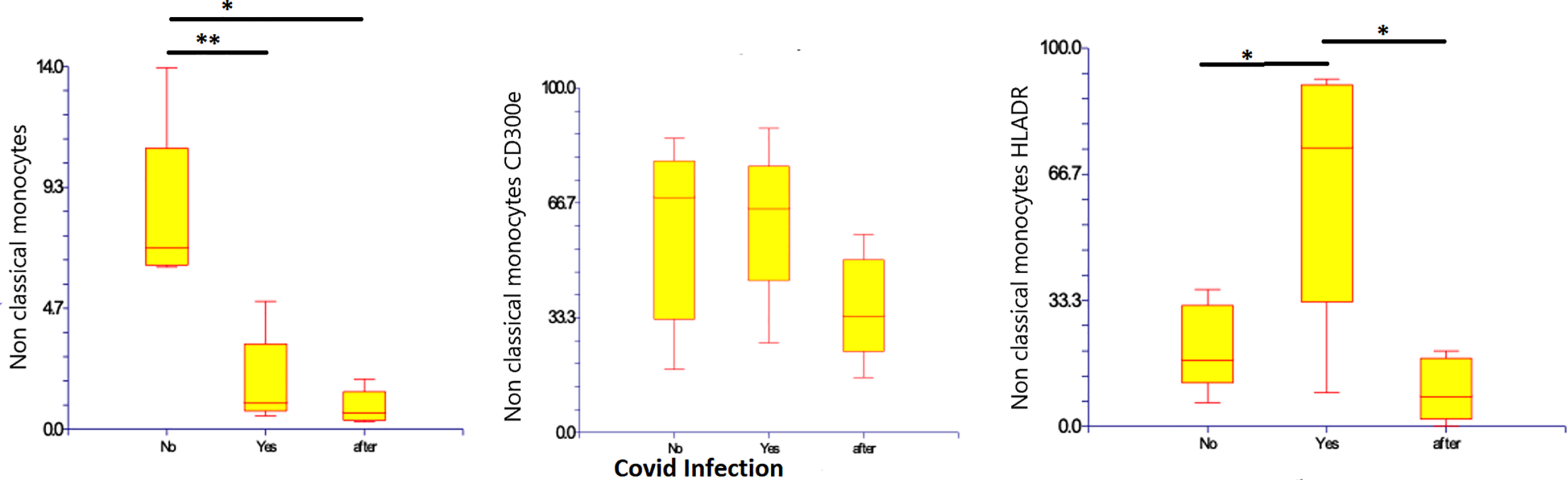
Intermediate CD14+ CD16+ subsets of monocytes, expression of percentage and expression of CD 300e and HLA-DR on intermediate CD14+ CD16+ subsets of monocytes before, during and after COVID-19 infection. Results are presented in box plots indicating the median as the horizontal line, 25^th^-75^th^ percentiles as the group distributions as boxes and 2.5-97.5% cumulative frequencies as whiskers. Outliners [identified by the 1.5 x inter-quartile range (IQR) criterion] are plotted as empty squares. * represents p<0.05 and ** represents p<0,01.

Intermediate monocytes seem to be increased in patients with Covid-19, representing 37% (8.2– 80.4%) of monocytes as compared to those with non-infected ESKD, at 11.2% (7.6 – 19.2% of monocytes). The population decreased at 10 months after recovery: 6.3% (5.3 – 8%) of monocytes (*p*: 0,049) compared to non-infected patients. The pro-inflammatory marker HLA-DR was more expressed on intermediate monocytes with Covid-19, at 80.2% (55.1 – 98.8%) as compared to non-infected ESKD patients at 68.7% (57.2-77.2%). Expression of CD300e was not significantly changed: 95.2% (89.2 – 98.8%) of intermediate monocytes compared to 96.2% (91.5– 100%) of intermediate monocytes before Covid-19 infection. At 10 months after recovery, the expression of HLA-DR intermediate monocytes increased significantly: 87.8% (82.8–93.4%) of intermediate monocytes (*p*:0,012) compared to non-infected patients. (**Table 6**) (**Figure 7**)

**Figure 8:**
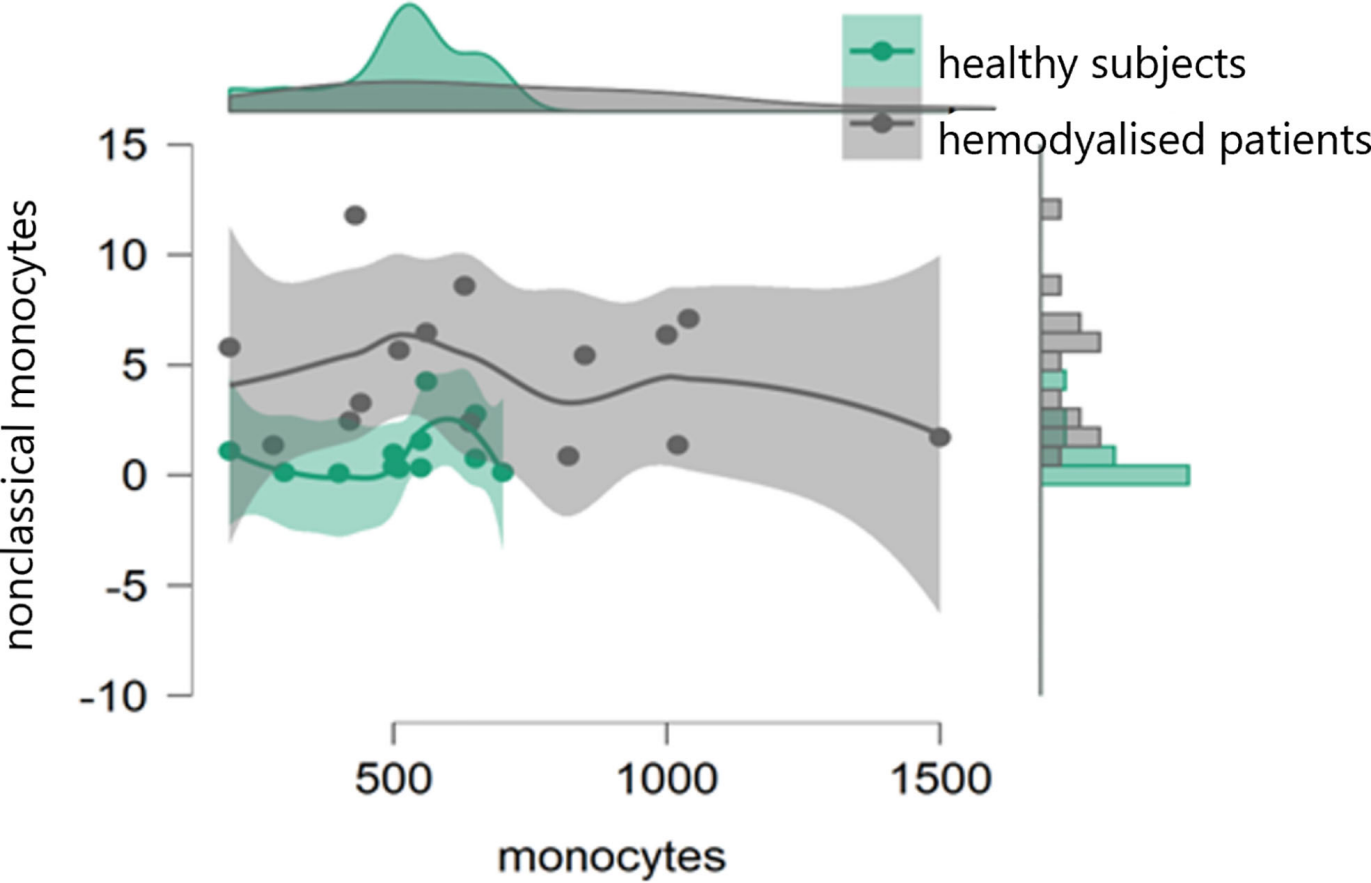
FACS plots histograms representing the monocytes subtypes before, during and after COVID infection. Acquisition on Gallios Beckman Coulter cytometer, gating was done with CD64 on monocytes and CD14/CD16 graph was used to discriminate monocytes subsets.

FACS dot plots with monocytes subtypes are represented in **Figure 8**, showing the differences before, during and after COVID-19 infection in study patients.

Surprisingly, we observed a prolonged immune effect of mild Covid 19 that was still significant after 10 months, although no other clinical complications, infections, thrombotic events, or cardiovascular events, were observed.

Using Spearman’s rho correlation, we obtained a positive correlation between the number of leucocytes and lymphocytes (*p*:0.017), and the number of monocytes and classical monocytes before Covid-19 infection. Also, between the number of lymphocytes and monocytes during Covid-19 infection (*p*:0.017), and between the number of leucocytes and monocytes (*p*:0,017), the number of leucocytes, and intermediate monocytes (*p*:0.017) and the number of monocytes and intermediate monocytes (*p*:0.017) after Covid-19 infection.

## Discussion and conclusion

Mortality from Covid 19 has been shown to be high in those with ESKD; renal failure is a risk factor for severe forms of Covid-19 infection (39, 42). Thus, we tried to determine if these patients treated by hemodialysis had different immune response regarding monocyte cells.

This is the first prospective study performed on ESKD patients that has focused on the effect of Covid-19 on monocyte subsets. Fortunately, all our patients only had mild-medium severity Covid.

In medium-severity Covid-19 infection, our results showed a significant decrease in the absolute number of lymphocytes, as has been already seen in severe Covid-19 (20, 43) (**Figure 4**) This suggests that ESKD immunity was more susceptible to the SARS CoV-2 virus, possibly because of chronic inflammation caused by the initial disease and periodical contact with the dialysis membrane. Fortunately, the immune effect was not related to Covid-19 severity and none of our patients had severe Covid. There was 100% survival at approximately one year after Covid infection, without secondary sequelae of the disease. The number of monocytes was not changed: this is in contrast to another study (Qin S. et al., 2020) that described a significant increase in the medium-severity forms of Covid-19 (44).

HLA-DR expression on monocytes is decreased in severe prolonged sepsis and is correlated with immune deficiency in intensive-care patients. However, our results show that this was not the case in our patients, which agrees with their mild form of Covid-19 and their quick recovery.

CD14low CD16+ monocytes have been described to have a pro-inflammatory activity. This is in line with increased expression of CD300e as compared to classical monocytes. ESKD patients are known to have some level of chronic inflammation and it is not really a surprise that Covid-19, known to induce severe activation of innate immunity, had increased the level of inflammation. What is more surprising is the long-term persistence of this extra inflammation after recovery from Covid-19 in our patients. These patients had no symptoms, such as fever, muscle, or joint pain after recovery, but had raised background inflammation which is known to lead to long-term cardiovascular pathology (4). This suggests that chronic inflammation must be considered in ESKD patients that have recovered from Covid-19.

In our study, we focused on a medium form of Covid-19 infection regarding changes in monocyte activation and subset expansion in patients with advanced-stage renal failure and on hemodialysis. We compared our results with those of other studies (reported within the last years) of patients with a medium form of Covid-19 infection but no renal failure. The advantage of our study is that we had controls without Covid that did not have significant changes.

HLA-DR and CD300e expression was analyzed by the percentage expression in each subset of monocytes that we considered more relevant than the nominal elevation or decreasing tendency in those subsets of monocytes. Our results show a slow decrease in the percentage of classical monocytes, an increase in intermediary monocytes, and a significant decrease of non-classical monocytes: this concurred with another study (Haschka D. et al, 2022) that reported depletion of non-classical monocytes and expansion of the intermediate subset (45). A study published in 2021 (Boumaza A. et al, 2021) reported that Covid-19 patients had decreased percentages of all subclasses of monocytes: classical, intermediate, and non-classical (43). Another study (Gatti A. et al, 2020) described a higher percentage of intermediate and non-classical monocytes associated with moderate-infection Covid compared to controls, and a significant decrease in non-classical and intermediate monocytes in severe cases (46). An increase in non-classical monocytes associated with severe forms of Covid-19 was also described in 2021 (Roussel M. et al, 2021) (47).

Percentage of non-classical monocytes was decreased with a higher expression of HLA-DR and mild elevation of CD300e (**Figure 5**) even though other reports show decreased expression of HLA-DR in patients with a medium form of Covid-19 infection and without advanced renal failure (Boumaza A. et al, 2021) (43). The percentage of intermediate monocytes were elevated (**Figure 7**) and comparable with other reports on patients with a medium form of Covid-19 infection and no advanced renal failure (Gatti A. et al, 2020) (46). However, we also found elevated expression of HLA-DR and lower expression of CD300e, which contradicts some reports that show decreasing expression of HLA-DR in patients with a medium form of Covid-19 infection but no advanced renal failure (Boumaza A. et al, 2021) (43). Other studies report a decrease in monocytes HLA-DR expression in severe forms of Covid-19 (Gatti A. et al, 2020, Qin S. et al, 2020) (44, 46, 48) and propose that the decrease of intermediate and non-classical monocytes and the downregulation of monocyte HLA-DR are indicators from severe forms of Covid-19 (Gatti A. et al, 2020) (46).

Overall, we found a tendency for increasing of HLA-DR and CD300e expression in some patients with medium-form Covid-19 infection and no advanced renal failure, which is similar to other reports (Zenarruzabeitia et al., 2021, Schulte-Schrepping J. et al., 2020) (20, 49). We can consider that advanced renal failure with hemodialysis does not change the inflammatory response against SARS-Cov2 in patients with medium-form Covid-19 infection.

When we analyzed the subsets of monocytes in patients with a medium form of Covid-19 infection and advanced renal failure we found a different distribution of inflammatory response: there was a decrease in classical and non-classical monocytes, as occurs in patients with severe forms of Covid, but apparently without worsening the outcome. We noticed also that non-classical monocytes expressed higher level of HLA-DR in our patients compared to patients with medium-form Covid-19 and no advanced renal failure (Boumaza A. et al, 2021) (43). Intermediary monocytes increased similarly in patients with medium-form Covid-19 infection and no advanced renal failure (Gatti A. et al, 2020) (46). The same elevated expression of HLA-DR was found in this subset, in contrast to patients with different forms of Covid-19 infection with no advanced renal failure: in other study expression of HLA-DR was decreased in non-classical and intermediate monocytes and was not related to the severity of Covid-19 (Boumaza A. et al, 2021) (43). These changes could suggest higher antiviral activity in non-classical monocytes and higher inflammatory and phagocytic activity in intermediate monocytes in patients with advanced renal failure and that have had a medium form of Covid-19 infection. If we also consider the results after recovery from Covid infection, it seems that proinflammatory monocyte status continued for many months afterwards.

In conclusion classical monocytes seem to be less affected during Covid-19 infection and there was no statistical evidence for their enhanced activity during or after Covid infection.

A decrease in non-classical monocytes during Covid infection was statistically significant, but the expression of HLA-DR increased leading to greater inflammatory activity. Furthermore, the percentage of non-classical monocytes continued to decrease and was less activated after Covid-19 infection when compared with data taken during or even before infection so we observed a persistent decrease in non-classical monocytes accompanied by the loss of inflammatory activity due to Covid infection.

Like non-classical monocytes, the percentage of intermediary monocytes decreased after a Covid-19 infection compared to before infection, but they retained inflammatory activity and a significant increase in HLA-DR expression compared to before infection and this is the most important result of our study because this class of intermediary monocytes is associated with increased cardiovascular mortality and morbidity in patients with ESKD. It seems that Covid-19 infection acts like a trigger to activate them after recovery from a Covid infection.

We need to see if this inflammatory status persists, what its consequences are on comorbidities and cardiovascular events, and how this can influence outcomes, morbidity, and mortality of these patients.

Finally, we conclude that ESKD associated with medium severity form of COVID-19 infection has a different evolution compared to those without renal damage, and associate a persistent inflammatory status related to monocyte behavior, and possible higher risk for cardiovascular complications long time after the infection.

## Data availability statement

The original contributions presented in the study are included in the article/supplementary material. Further inquiries can be directed to the corresponding author.

## Ethics statement

The studies involving human participants were reviewed and approved by University Emergency Hospital Bucharest, Emergency Clinical Hospital Bucharest. The patients/participants provided their written informed consent to participate in this study.

## Author contributions

VB has done the design of study, database, statistical analysis and interpretation of results, discussion and conclusions LA, LR and LD have done the database HB has done the interpretation of immunophenotypical results and submit the manuscript ID did the immunophenotypical acquisition and analysis CL revised the manuscript especially the results and discussion part A-MV approved the final version of manuscript. All authors contributed to the article and approved the submitted version.
